# Comparative Assessment of Different Yeast Cell Wall-Based Mycotoxin Adsorbents Using a Model- and Bioassay-Based In Vitro Approach

**DOI:** 10.3390/toxins15020104

**Published:** 2023-01-24

**Authors:** Ran Xu, Alexandros Yiannikouris, Umesh K. Shandilya, Niel A. Karrow

**Affiliations:** 1 Department of Animal Biosciences, University of Guelph, Guelph, ON N1G2W1, Canada; 2 Alltech Inc., Center for Animal Nutrigenomics and Applied Animal Nutrition, 3031 Catnip Hill Road, Nicholasville, KY 40356, USA

**Keywords:** adsorption, gastrointestinal digestion model, isotherm models, in vitro cell culture, liquid chromatography, novel detoxification strategies, remediation, ruminants, sequestration, toxicity

## Abstract

Frequently reported occurrences of deoxynivalenol (DON), beauvericin (BEA), and, to a lesser extent, ochratoxin A (OTA) and citrinin (CIT) in ruminant feed or feedstuff could represent a significant concern regarding feed safety, animal health, and productivity. Inclusion of yeast cell wall-based mycotoxin adsorbents in animal feeds has been a common strategy to mitigate adverse effects of mycotoxins. In the present study, an in vitro approach combining adsorption isotherm models and bioassays was designed to assess the efficacy of yeast cell wall (YCW), yeast cell wall extract (YCWE), and a postbiotic yeast cell wall-based blend (PYCW) products at the inclusion rate of 0.5% (*w/v*) (ratio of adsorbent mass to buffer solution volume). The Hill’s adsorption isotherm model was found to best describe the adsorption processes of DON, BEA, and CIT. Calculated binding potential for YCW and YCWE using the Hill’s model exhibited the same ranking for mycotoxin adsorption, indicating that BEA had the highest adsorption rate, followed by DON and CIT, which was the least adsorbed. PYCW had the highest binding potential for BEA compared with YCW and YCWE. In contrast, the Freundlich isotherm model presented a good fit for OTA adsorption by all adsorbents and CIT adsorption by PYCW. Results indicated that YCW was the most efficacious for sequestering OTA, whereas YCWE was the least efficacious. PYCW showed greater efficacy at adsorbing OTA than CIT. All adsorbents exhibited high adsorption efficacy for BEA, with an overall percentage average of bound mycotoxin exceeding 60%, whereas moderate efficacies for the other mycotoxins were observed (up to 37%). Differences in adsorbent efficacy of each adsorbent significantly varied according to experimental concentrations tested for each given mycotoxin (*p* < 0.05). The cell viability results from the bioassay using a bovine mammary epithelial cell line (MAC-T) indicated that all tested adsorbents could potentially mitigate mycotoxin-related damage to bovine mammary epithelium. Results from our studies suggested that all tested adsorbents had the capacity to adsorb selected mycotoxins in vitro, which could support their use to mitigate their effects in vivo.

## 1. Introduction

Mycotoxins are toxic secondary metabolites naturally produced by various fungal species such as *Aspergillus, Fusarium,* and *Penicillium* spp. Different mycotoxins naturally co-contaminate a variety of agricultural commodities of plant origin, including cereal grains and forages, under favorable environmental conditions; thus, they are often detected in ruminant feed or feedstuffs [1,2], which poses a significant threat to animal performance and health as well as food safety. Consumption of mycotoxin-contaminated feed by animals can lead to a variety of adverse effects, such as compromised productivity and fertility [2,3] and increased susceptibility to infectious diseases [4,5,6], which contribute to important economic losses worldwide. It has been projected that weather conditions associated with climate change will exacerbate mycotoxin contamination [7,8]. The problem is further complicated by global trading of feed ingredients and accessibility issues of an agriculture system under stress. 

Certain mycotoxins also have human health implications due to their carry-over into animal-derived by-products. Ruminant milk, for example, has been reportedly contaminated with certain mycotoxins such as ENB, OTA, and aflatoxin M1 [9,10,11]. This could be attributed to a modulation of factors influencing mycotoxin toxicokinetics, such as the mycotoxin biodegradation capabilities of the rumen microflora [12]; the recirculation of certain toxin via enterohepatic circulation [13,14]; increased mycotoxin presence in the rumen due to the increasingly high exposure in feed or feedstuff [15]; and effectiveness of the hepatic biotransformation metabolic process [16,17]. All these factors influence the resulting toxin biodistribution throughout the animal, resulting in their possible subsequent circulatory transport to animal mammary glands.

A variety of pre- and post-harvest approaches have been implemented to manage mycotoxin contamination and mitigate their negative effects on animals [2], among which inclusion of mycotoxin adsorbents in animal feeds as a “final-stage-control” has been widely used by the feed industry due to its economic feasibility [2,18]. These mycotoxin adsorbents could mitigate the bioavailability of mycotoxins in the gastrointestinal tract of animals by forming mycotoxin–adsorbent complexes that are consequently excreted via the fecal route, thus reducing mycotoxin uptake and potential distribution to the blood and target organs [19]. As an alternative to inorganic clay-based adsorbents that have limitations, such as limited and specific interactions with mycotoxins, possible contamination with other deleterious compounds (polychlorinated biphenyls, dioxins, and heavy metals), and high inclusion rate in feed when used as a mycotoxin sequestrant, organic adsorbents such as yeast cell wall-based adsorbents have been developed and represent a more efficient [20] and safe product substitute [21]. Generational improvements have complemented those formulations with functional carbohydrate and/or nutritional add-ons. However, their efficacy varies depending on different product compositions and mycotoxins types [18,22,23,24,25,26]. “Emerging mycotoxins”, which represent lesser-known or newer forms of mycotoxins (this currently includes toxins such as beauvericin, enniatins, moniliformin, alternariol, and phomopsins), have become a center of interest due to their occurrence and potential concerns for animal production systems [15,27,28,29]. With an evolving mycotoxin focus, adsorbent products are also being investigated for enhancing their adsorption efficacies [30,31] as well as improving and extending their specificity, which in turn requires new assessment methodologies to be carried out in a timely and cost-effective manner to characterize novel product attributes.

A scientific report submitted to the European Food Safety Authority (EFSA) [19] suggested that in vitro analysis of mycotoxin–adsorbent interactions is a powerful tool for screening, identifying, and ranking potential mitigation solution and that it offers high throughput, cost effectiveness, and potential predictability of efficacy toward an animal in vivo application. In vitro models combining gut simulation and cell culture bioassays could not only closely mimic gastrointestinal conditions that are physically comparable to in vivo conditions but may also account for the dynamic interactions among mycotoxins, adsorbents, and cells [19]. Mammary epithelial cells are one of the critical functional cellular components of the mammary gland which work to maintain homeostasis critical for MG functionality [32,33]. Bovine udder health is important when high-yield production of high-quality milk is considered.

The aim of this work was to assess the multi-mycotoxin adsorption mitigation efficacy of several yeast cell wall-based mycotoxin adsorbent products using an in vitro gastrointestinal digestion/bovine mammary epithelial cell system that combines both adsorption isotherm models and bioassays. Three yeast cell wall-based mycotoxin adsorbent products were tested, which were classified as yeast cell wall (YCW), yeast cell wall extract (YCWE), and a postbiotic yeast cell wall-based blend (PYCW). Four mycotoxins—deoxynivalenol (DON), beauvericin (BEA), ochratoxin A (OTA), and citrinin (CIT)—were selected for their significance based on public concern, their occurrence in ruminant feed or feedstuff [1,34,35,36,37,38], their known toxic effects [23,39,40,41], and their agri-economic significance [2,42]. The present study not only sheds light on the potential multi-mycotoxin interaction capacity of the tested binder products, especially for novel adsorbent compositions such as PYCW, but also provides insight into BEA and CIT mitigation, which has not been widely studied. 

## 2. Results

### 2.1. The Goodness of Fit of Adsorption Isotherm Models

The goodness-of-fit index was obtained to assess the best-fitting adsorption isotherm model for each mycotoxin- and adsorbent-specific process with the intent to predict maximum adsorption capacity, affinity, intensity, and cooperativity of the adsorption process, which characterize the nature of the interaction. Our results indicated that the Hill’s model was the best-fitting model to describe most of the yeast cell wall-based adsorbent–mycotoxin complexation processes studied herein. This model was defined by a high coefficient of determination R^2^, low residual sum of squares SS_RES_, and low reduced chi-squared χ^2^ values (Table 1). The Freundlich model appeared to fit well with the sequestration data produced by PYCW for OTA and CIT. Moreover, the Hill’s and Freundlich models were comparable in terms of describing the interactions of DON-YCW and OTA-YCWE. Adsorption of OTA by YCW fit with Hill’s and Langmuir isotherm models to a comparable extent. However, none of the three adsorption isotherm models were able to describe the nature of the adsorption process of DON to PYCW in the present study. 

**Figure 1:**
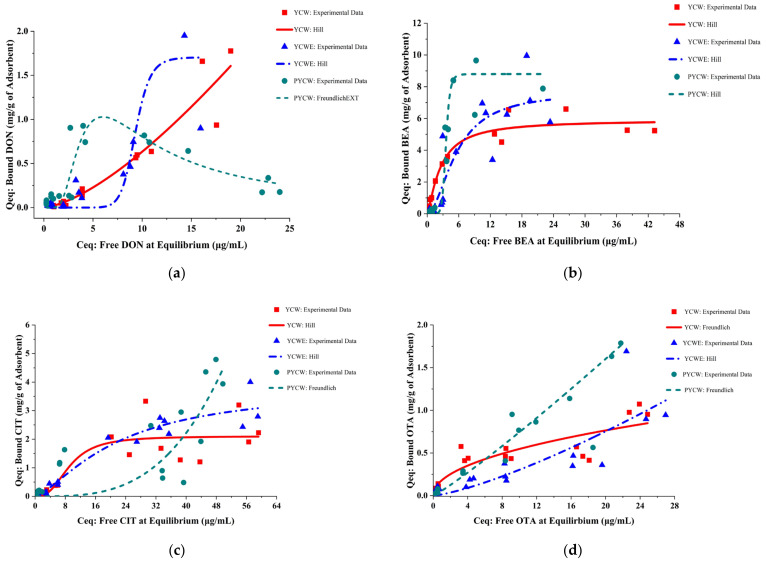
Equilibrium adsorption isotherms for each tested adsorbent (yeast cell wall (YCW), yeast cell wall extract (YCWE), and postbiotic yeast cell wall-based blend (PYCW)) at 0.5% (*w/v*) toward each tested mycotoxin: (**a**) deoxynivalenol (DON); (**b**) beauvericin (BEA); (**c**) citrinin (CIT); and (**d**) ochratoxin A (OTA). The isotherms were obtained by fitting the experimental data to selected best-fitting models. The symbols represent experimental data, and the lines are the model fit to each experimental dataset.

The adsorption isotherms of the selected best-fitting model for each adsorption process were obtained by plotting Q_eq_ against C_eq_ [19,43] using OriginPro 2022, and the results are presented in Figure 1. Although the complexation of DON by PYCW was poorly fitted to all three models, it was best described with FreundlichEXT (Equation (1)) [44,45], a built-in extended Freundlich model in OriginPro (R^2^ = 0.7032; SS_RES_ = 0.555; reduced χ^2^ = 0.037). The adsorption isotherm is presented in Figure 1a.

(1) $$y=ax^{bx^{-c}}$$

where *y* = Q_eq_; *x* = C_eq_; and *a*, *b*, and *c* are the parameters.

### 2.2. Isotherm Parameters of Best-Fitting Models

#### 2.2.1. Hill Isotherm Model Fitting and Binding Potential

Isotherm parameters obtained from the best-fitting model are presented in Table 2. The Hill’s isotherm model was applied with the assumption that the adsorption process is a cooperative phenomenon when *n* ≠ 1, which means that adsorbates have the ability to bind at one site on the adsorbent, which in turn could influence other binding sites on the same adsorbents [46]. When *n* > 1, binding has positive cooperativity, meaning binding of a mycotoxin molecule increases the affinity and chance for another molecule to be bound. Conversely, when *n* < 1, negative cooperativity occurs, where binding of a mycotoxin molecule decreases affinity and further makes the binding of other molecules less likely [46,47].

Our results from the Hill’s model fitting indicated that adsorption of DON and CIT by YCW and YCWE as well as adsorption of BEA by all three tested adsorbents had a positive cooperativity. Specifically, sequestration of DON by YCWE exhibited the most pronounced cooperative binding, and YCWE had the most binding sites available for DON (*n* = 12.46 ± 1.258). In contrast, cooperative binding was the least pronounced with the adsorption of BEA by YCW, and YCW had the fewest binding sites available for BEA, which accounted for the smallest *n* value (*n* = 1.30 ± 0.242).

We next calculated the binding potential, the ratio of maximum mycotoxin update to dissociation constant per site for the mycotoxin–adsorbent interaction that were best fitted with the Hill’s model, as another endpoint to assess adsorption efficacy. As shown in Table 3, mycotoxin binding potential ranked BEA > DON > CIT for both YCW and YCWE. For the sequestration of BEA, PYCW exhibited the highest binding potential compared with YCW and YCWE.

#### 2.2.2. Freundlich Isotherm Model Fitting

As shown in Table 2, parameters obtained from Freundlich model fitting indicated that YCW possessed the greatest sequestration efficacy toward OTA, with the highest K_f,_ and lowest 1/n (highest n) values of 0.18 and 0.48, respectively. In contrast, YCWE was the least efficacious at interacting with OTA as indicated by the lowest K_f_ and *n* values. The results also indicated that PYCW exhibited greater efficacy at adsorbing OTA than CIT.

### 2.3. Adsorption Efficiency

**Figure 2:**
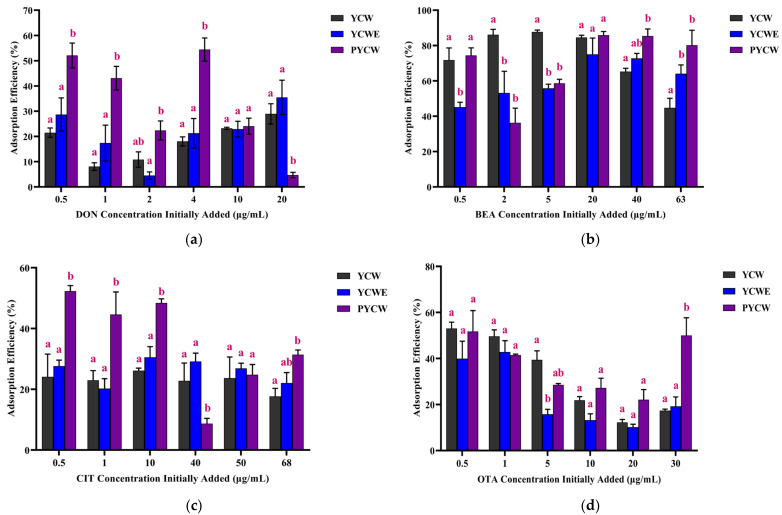
Adsorption efficiency of the tested adsorbents (yeast cell wall (YCW), yeast cell wall extract (YCWE) and postbiotic yeast cell-wall-based blend (PYCW)) at 0.5% (*w/v*) toward different concentrations of (**a**) deoxynivalenol (DON), (**b**) beauvericin (BEA), (**c**) citrinin (CIT), and (**d**) ochratoxin A (OTA). Results are the mean ± SEM of three independent experiments. ^a–b^ Different letters indicate significant differences between adsorbent treatments within a given mycotoxin concentration (*p* < 0.05).

The adsorption efficiency was also assessed by calculating the percentage of adsorbed mycotoxins. As shown in Figure 2a, Overall DON, at concentrations lower than 10 μg/mL, was better adsorbed by PYCW than other adsorbents, whereas PYCW had a lower efficacy for sequestering DON at higher concentrations (> 20 μg/mL). There were no significant differences in the efficacy of YCW and YCWE at adsorbing DON within the concentration range tested in the present study (*p* > 0.05). For BEA (Figure 2b), PYCW and YCWE exhibited identical overall sequestration efficacies (*p* > 0.05), except for at 0.5 μg/mL of BEA, where PYCW was better than YCWE (*p* < 0.05). YCW appeared to be more efficacious than PYCW at adsorbing BEA at its lower concentrations, whereas YCW was found to be less efficacious than PYCW at adsorbing higher concentrations of BEA (*p* < 0.05). 

Figure 2c indicates that PYCW adsorbed the highest amount of CIT at concentrations less than 40 μg/mL (*p* < 0.05), but it was the least efficacious at 40 μg/mL (*p* < 0.05). Both YCW and YCWE exhibited similar adsorption of CIT (*p* > 0.05). Overall, all three adsorbents demonstrated the same sequestration properties for OTA (*p* > 0.05); however, YCW was better than YCWE for OTA adsorption at 5 μg/mL (*p* < 0.05). PYCW had better sequestration properties than the two other adsorbents at the highest concentration of OTA (30 μg/mL) (*p* < 0.05) (Figure 2d).

The overall adsorption efficiency was calculated by averaging the adsorption efficiency across all tested concentrations for a given mycotoxin, and the results are presented in Table 3. Our results demonstrated that all tested yeast cell wall-based adsorbents were capable of binding DON, BEA, CIT, and OTA at ranges of 18.45–33.46%, 61.00–73.37%, 22.90–35.04%, and 23.55–36.86%, respectively. Specifically, PYCW exhibited the best binding properties toward DON and was also more efficacious at sequestering CIT and OTA than YCW and YCWE, respectively (*p* < 0.05). YCW adsorbed BEA and OTA more efficaciously than YCWE (*p* < 0.05). All three adsorbents exhibited a high capacity for binding BEA compared to the other mycotoxins, as indicated by the percentage of adsorption greater than 60%.

### 2.4. Bioassay Adsorbent Efficacy Assessment

Bioassays were also performed to evaluate absorbent efficacy by quantifying the toxic effects of residual free mycotoxins in the supernatant using MAC-T cell viability as an endpoint; higher cell viability was suggestive of a lower amount of residual mycotoxin in the supernatant and, therefore, higher mitigation efficacy of the tested adsorbent.

**Figure 3:**
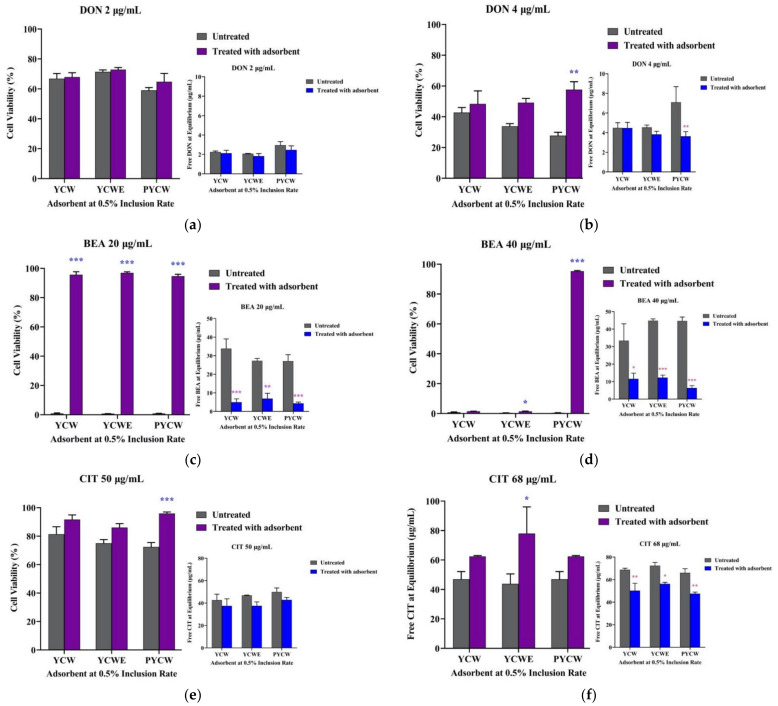

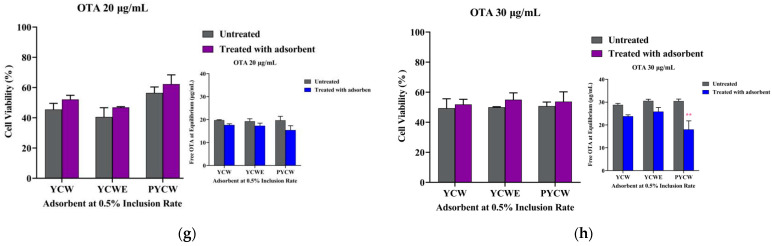
Residual concentrations of each mycotoxin in the supernatant measured by LC-MS analysis and their effects on MAC-T cell viability in the presence of each tested adsorbent (yeast cell wall (YCW), yeast cell wall extract (YCWE), and postbiotic yeast cell-wall-based blend (PYCW)) at 0.5% (*w/v*). (**a**,**b**) Deoxynivalenol (DON) initially added at 2 and 4 μg/mL, respectively; (**c**,**d**) beauvericin (BEA) initially added at 20 and 40 μg/mL, respectively; (**e**,**f**) ochratoxin A (OTA) initially added at 20 and 30 μg/mL, respectively; (**g**,**h**) citrinin (CIT) initially added at 50 and 68 μg/mL, respectively. Values are presented as the mean ± SEM of three independent experiments. Significant differences compared to control groups without adsorbent treatment are indicated at *p* < 0.05 (*), *p* < 0.01 (**), and *p* < 0.001 (***).

Our results (Figure 3) indicated that at 0.5% inclusion rate, the cytotoxic effect of DON at 4 μg/mL, BEA at 40 μg/mL, and CIT at 50 μg/mL (*p* < 0.05) were significantly lower with addition of PYCW. Inclusion of YCWE also resulted in significantly lower toxic effects on MAC-T cells with 20 and 40 μg/mL of BEA as well as 68 μg/mL of CIT (*p* < 0.05). The toxic effects of 20 μg/mL of BEA on cell viability were also markedly reduced with inclusion of YCW. The biochemical assay LC-MS-based analytical results were overall consistent with the bioassay analysis and confirmed the higher cell viability observed in the adsorbent-treated groups compared to control groups without inclusion of adsorbents in the presence of the tested mycotoxins, which was the subsequent result of reduced residual mycotoxin concentrations found in the supernatant of treated cells (Figure 3).

## 3. Discussion

Mycotoxins are naturally produced by filamentous fungi as coping strategies under environmental stress conditions to enhance fungal pathogenicity, virulence, and aggressiveness [48]. Therefore, mycotoxins as natural contaminants are inherent and unavoidable problems, and they have become one of the most significant hazards in feed and livestock industries [2]. The frequent detection of DON, BEA, CIT, and OTA in ruminant feed or feedstuff could pose a potential threat to ruminants, especially those with a compromised ruminal ecosystem that could lead to insufficient biodegradation of mycotoxins by the rumen microflora, thus allowing the mycotoxins to bypass the rumen compartment to reach distal portions of the digestive system. This in turn could result in an increase in their distribution to tissues such as the mammary gland. In recent European studies, DON was detected in maize silage with the highest concentration being greater than 300 μg/kg [49]. Additionally, BEA was found in silage samples with a maximal concentration of 214 µg/kg [1]. The highest concentrations of OTA and CIT were reported to be 305.6 μg/kg in dairy cattle feed and 81 μg/kg in samples of Canadian forage for dairy cattle and goats, respectively [36,37].

Addition of yeast cell wall-based mycotoxin adsorbents to animal feeds has been part of the widely used integrated mycotoxin management approach to mitigate potential negative effects of mycotoxins on animals [2]. The inner layer of yeast cell wall insoluble polymeric β-glucans constituents is composed mostly of long chain of β-(1,3)-D-glucans decorated with side chains of β-(1,6)-D-glucans and arranged in helical structures. They have been identified as key functional actors responsible for sequestering mycotoxins through interactions between mycotoxin chemical functional groups and hydroxyl-groups present on each monomer of the β-glucopyranosyl moiety forming those chains via hydrogen bonding and van der Waals electrostatic interactions [18,50]. Branched β-(1,6)-D-glucans connect to N-acetylglucosamine polymeric chains that form chitin, a minor component of the inner cell wall that contributes to the insolubility of the overall architecture of β-glucans as well as the outer cell wall composed of mannoproteins [51]. The yeast cell wall carbohydrate network organization also contributes to its resistance to digestion and to maintaining its binding capacity throughout the course of the gastrointestinal tract in animals while preserving its dynamic properties and cell wall integrity [18]. Other bioactive components included in the formulation of the tested adsorbent products, such as algal components, vitamins, and postbiotic fatty acids, could further mitigate mycotoxin-related negative effects on animals by various biological activities, such as antioxidant properties, supporting intestinal health, antimicrobial activities, and counteracting the immunomodulatory effects of mycotoxins [22,23]. Algal components could also have the potential to directly enhance the efficacy of the adsorptive process complementary to yeast cell wall β-glucans by providing additional cell wall polysaccharides as binding sites for mycotoxins, which has found other applications such as biosorbents used for the removal of environmental pollutants [52].

In the present study, the adsorption experiment was conducted in vitro under conditions simulating the temperature and pH of consecutive gastric and intestinal digestion steps occurring in the dairy cattle gastrointestinal tract. A 4 h set incubation time was implemented to simulate the passage time through the gastrointestinal tract. It was also anticipated to provide sufficient contact time for adsorption to reach equilibrium based on previous studies [24,53,54]. Our group previously reported that DON, BEA, OTA, and CIT decreased cell viability of MAC-T cells [41,55]. Coupled with an adsorption isotherm model-based approach, a MAC-T cell-based bioassay was used as an in vitro model of bovine mammary epithelium to investigate the cytotoxic effects of residual mycotoxin concentrations present in the supernatant and their impact on cell viability with or without the inclusion of three yeast cell wall-based mycotoxin treatments tested individually. The outcome was used as an indicator of adsorption efficacy following in vitro incubation simulating ruminant GIT conditions.

The three adsorption isotherm models used in our study—Hill’s, Langmuir, and Freundlich—are well-established models widely used in the field of mycotoxin research to study adsorption of a variety of mycotoxins, including the ones investigated herein as well as other toxins such as aflatoxin B1, fumonisin B1, zearalenone, T-2 toxin, and patulin, as previously reported in the literature [43,47,50,56,57]. They were used to describe adsorbent efficacy and to provide an understanding of the physicochemical mechanisms of the adsorption process. We found that the Hill’s adsorption isotherm model best described DON, BEA, and CIT adsorption processes by the tested binders. In contrast, the Freundlich model appeared to fit well with the sequestration data of OTA by all tested adsorbents as well as the sequestration data of CIT by PYCW. Our results indicated that the goodness of fit of different adsorption isotherm models varied and could depend on factors such as the types of mycotoxins and adsorbents, which was also observed in previous studies [18,53,57,58,59]. The Hill’s model appeared to provide the best description for most mycotoxin–adsorbent interactions tested in the present study, which was consistent with and confirmed previous findings in the literature on the appropriateness of Hill’s adsorption isotherm model for assessing efficacy of organic adsorbents for sequestering mycotoxins [18,53,57,59].

The positive cooperativity of the interaction between the mycotoxin and yeast cell wall-based adsorbents highlighted by the Hill’s model in the present study accounted for the dynamicity of the parietal structure, as it indicated that the first sets of mycotoxin molecules binding the macrostructure induced further conformational changes of the cell wall, resulting in an increase of the number of further mycotoxin molecules to be bound. As previously reported, the three-dimensional conformational mobility of the yeast cell wall could play a critical role in this adsorption process [25,50,60]. In contrast, all OTA adsorptive processes and CIT-PYCW interactions were best described by Freundlich isotherm model. Our results suggested that these adsorption processes occurred on a heterogenous adsorbent surface with an exponential distribution of active sites and energy [46].

On the other hand, the DON–PYCW interaction was not well described by any of the other three selected models used in this study. Instead, it was adequately fitted into the FreundlichEXT model, and the relationship exhibited a biphasic isotherm shape. This could suggest that a competitive adsorption had occurred where other components in the adsorbate–adsorbent system could compete with further attachment of DON molecules to the active sites on PYCW [61,62]. This could result in a lower efficacy of PYCW at high DON concentrations as used in the present study. However, we acknowledge that the mycotoxin concentrations used in the present studies were higher than the levels commonly encountered by ruminants through naturally contaminated feed, although global climate change is likely to increase the occurrence of mycotoxin contamination in the future. The aim of applying this in vitro model was to perform an initial screening of adsorption efficacy of the tested products towards multiple mycotoxins in a relatively large-scale and high-throughput manner and to establish a complete kinetic of interaction.

We next calculated “binding potential” using obtained parameters for the Hill-fit adsorption processes to predict the adsorption efficacy. Binding potential is a concept and an outcome measurement widely used in radioligand binding studies that are based on equilibrium binding studies [63,64]. Similarly, a comparable concept with a similar mathematical formula “affinity rate” has also been proposed in previous studies for mycotoxin adsorption [47,50]. Our results, as demonstrated by other scientific studies [20,50,65], confirmed that a given adsorbent could have varying efficacy towards different mycotoxins, which could be attributed to the wide range of molecular stereochemistry and chemical characteristics of the mycotoxin under consideration; major parameters include pKa, water solubility, polarizability, topological surface area, molecular weight (100 to 1000 Da), rotatable bonds, number and position of hydrogen donor sites contributing to the overall electrostatic interaction, and hydrophobic and π-stacking effects promoted by solute interactions. In addition, dynamicity of the carbohydrate composition of the product could contribute to changes in the number of sites of interaction depending on the concentration of the mycotoxins present [50] and the three-dimensional conformation of this network and associated binding sites, as determined in binding kinetic studies and in molecular mechanics and dynamic simulations [25,60]. All these parameters could contribute to the overall interaction efficacy of an adsorbent to a given mycotoxin.

We also assessed adsorption efficiency of each adsorbent at different concentrations of each mycotoxin as well as the overall mean adsorption efficiency. We observed that efficacy of different adsorbents could vary depending on the concentrations of a given mycotoxin. Such efficacy differences in the present study could suggest that the optimal initial mycotoxin-concentration-to-adsorbent-dosage ratio could vary depending on the adsorbents and mycotoxins, which was previously reported to be one of the factor influencing the adsorption process through mass transfer [66,67,68]. The results for the overall mean adsorption efficiency also suggested differences in adsorbent efficacy for a given mycotoxin, which could be dependent on adsorbent-composition traits, which have previously been reported to include the origin of yeast, the strain, the percentage of cell wall components, the distribution of β-(1,6)-D-glucans and β-(1,3)-D-glucans, and the biomass density during the fermentation process [18,50].

The results produced from the bioassay indicated that inclusion of adsorbents statistically mitigated the adverse effects of selected mycotoxins on cell viability, which was further confirmed biochemically by lower free residual mycotoxin concentrations in the buffer environment. The overall consistency between bioassay and mycotoxin-quantitative results not only suggested the appropriateness of the cell-based approach for investigating the efficacy of organic yeast cell-wall-based adsorbents at sequestering certain mycotoxins but also suggested that the two approaches may have different sensitivities and could be used complementarily to provide a better understanding of adsorption efficacy in a biological context. Moreover, for the first time (to our knowledge), an emerging mycotoxin (BEA) was evaluated for its mitigation in the present study, demonstrating that yeast cell wall-based adsorbents have the capability to interact effectively with such a mycotoxin.

## 4. Conclusions

Cost-effective adsorbing agents with a multi-mycotoxin adsorption capacity are of significant importance for mitigating mycotoxin incidence in livestock considering the high costs associated with animal feeding and high co-occurrence of multiple mycotoxins. In the current study, a multi-endpoint in vitro approach combining chemical assays and bioassays was designed to assess the efficacy of three yeast cell wall-based mycotoxin adsorbent formulations to sequester DON, BEA, OTA, and CIT, which are mycotoxins that have been detected in ruminant feed and feedstuff that could have potential adverse effects on the bovine mammary gland. Our results indicated that all tested adsorbents were able to adsorb these selected mycotoxins to varying degrees. Results showed the benefit of the PYCW formulation related to DON sequestration and the overall efficacy of all three adsorbents at interacting with emerging toxins, which resulted in lower cytotoxicity. These findings support the use of yeast cell wall-based products to help mitigate mycotoxin exposure in vivo.

## 5. Materials and Methods

### 5.1. Chemicals and Mycotoxin Adsorbents

DON, BEA, OTA, and CIT (Sigma-Aldrich, St. Louis, MO, USA) were each dissolved in dimethyl sulfoxide (DMSO) to a stock concentration of 5 mg/mL, which were stored at −20 °C. Mycotoxin working solutions were prepared by diluting the stock solution to the designated concentrations as described below. Three yeast cell wall-based mycotoxin adsorbent products classified as yeast cell wall (YCW, Mycosorb^®^), yeast cell wall extract (YCWE, Mycosorb^®^A+) and postbiotic yeast cell wall-based blend (PYCW, Mycosorb^®^D+) were supplied by Alltech, Inc. (Nicholasville, KY, USA).

### 5.2. Mycotoxin Adsorption Experiment in Aqueous Buffer

The in vitro adsorption experiment was conducted in triplicate under conditions that attempted to mimic gastric and intestinal phases of the dairy cattle gastrointestinal tract (GIT) [69]. Three types of controls were included: a buffer solution without mycotoxin or adsorbent (vehicle control), a buffer solution with adsorbent but without mycotoxin (mycotoxin-free control), and a buffer solution with each mycotoxin at the designated concentrations but without an adsorbent (adsorbent-free control). The treatment groups contained both the selected adsorbent and individual mycotoxin. These controls and treatment groups were subjected to the same experimental procedure as described below where applicable. Each selected adsorbent (0.05 g) was weighed and suspended in a reaction tube containing 10 mL of ultrapure water (Milli-Q™, MilliporeSigma™, Burlington, MA, USA) to reach its final inclusion rate of 0.5% (*w/v*) [20,24,25]. The pH was then adjusted to 3 with 0.5 and 1M HCl to simulate gastric digestion [18,20,69]. Each of the following mycotoxins, DON (0.5, 1, 2, 4, 10, and 20 μg/mL), BEA (0.5, 2, 5, 20, 40, and 63 μg/mL), OTA (0.5, 1, 5, 10, 20, and 30 μg/mL), and CIT (0.5, 1, 10, 40, 50, and 68 μg/mL) was then added to the reaction system to reach six designated concentrations that spread over the widest possible concentration range provided in a scientific reported submitted to EFSA [19]. The concentration selection was also based off of the cell viability results from our previously published study [41]. The entire reaction for each sample was performed under constant agitation on a temperature-controlled orbital shaker maintained at 39 °C at 100 rpm for 2 h. After incubation, the pH was neutralized to 7 with NaOH (0.5 and 1M) to simulate intestinal digestion, and the samples were then further incubated for an additional 2 h under the same stirring and temperature conditions [18,24,70].

At the termination of the incubation process, samples were centrifuged at 4600× *g* for 25 min at 4 °C. Subsamples (500 μL and 1 mL) of the resulting supernatant were preserved at −20 °C until LC-MS and cell-based assay analyses, respectively.

### 5.3. Analytical Methodology for Mycotoxin Quantification

An ultra-performance liquid chromatography system (UPLC Acquity^®^ H-class, Waters Corp., Milford, MA, USA) interfaced with a high-resolution hybrid time of flight mass spectrometer (Vion™, ESI-IMS-QTOF-MSE, Waters Corp., Milford, MA, USA) was used to perform the quantification of mycotoxin analytes using a full scan mode accurate mass screening. The UPLC system was equipped with a CORTECS reverse phase C18 solid core UPLC column (2.1 × 100 mm, 2.7 μm particle size, Waters Corp., Milford, MA, USA) operated at 40°C under a flow rate of 0.42 mL/min. Samples were maintained in the autosampler at 4.0°C, and a volume of 10 μL per sample was injected. The VION™ mass spectrometer was fitted with an electrospray ionization source (ESI+), operated in positive and sensitivity modes, and set up for a survey scan for low (MS) and high energy (MSMS) experiments using a collision energy ramp from 25 to 65 eV and a mass acquisition range of *m/z* 50–1000 with a scan time of 0.5 s. Capillary voltage was set up at 3.00 kV, with a source temperature and desolvation temperature of 115 and 500 °C, respectively, using nitrogen as a cone and desolvation gas (50 and 600 L/h, respectively). Lock mass correction of the instrument during analysis and subsequent data processing was performed using a leucine-enkephalin (100 pg/μL) internal standard, which was automatically infused at 2 min intervals over the runtime period.

Analytes were individually separated using a dual mobile phase gradient consisting of LC/MS grade Optima^®^ water (Fisher Chemical, Fair Lawn, NJ, USA) and either methanol (DON analysis) or acetonitrile (Pharmco by Greenfield Global, Irvine, CA, USA, for OTA, CIT, and BEA analysis) amended with 0.1% formic acid (*v*/*v*) over an 8.0 min runtime. The elution time was 3.3, 4.2, 5.0, and 5.1 min for DON, OTA, CIT, and BEA, respectively. Quantification was performed using a six-point matrix match linear calibration curve (R^2^ > 0.99) of the four different analytes prepared in aqueous buffer media as described in Section 2.2. An aliquot of 10 μL of previously prepared samples (Section 2.2) were diluted in a solution of water:acetonitrile:formic acid (50:50:0.1%, *v*/*v*/*v*) in a final volume of 300 μL. DON samples were analyzed individually, whereas BEA, OTA, and CIT samples were mixed together before UPLC-ESI-IMS-QTOF-MSE analysis.

Data management was enabled by means of the UNIFI Scientific Information System v1.9.4.053 and the Vion™ IMS QTOF driver pack 2.2.0 (Waters Corp., Milford, MA, USA).

### 5.4. Equilibrium Adsorption Isotherms

In vitro mycotoxin sequestration experiments were based on the reversible equilibrium binding reaction between adsorbents and free mycotoxins to form a bound mycotoxin–adsorbent complex [19]. Adsorption isotherms have been an efficient tool to evaluate mycotoxin-detoxifying agents [19]. They describe equilibrium performance and interaction mechanisms of the adsorbate and adsorbent, which take into consideration both equilibrium data and adsorption properties [46]. Equilibrium adsorption isotherm models were fitted with the analytical data generated by mass spectrometry to assess the efficacy of each individual adsorbent to sequester each studied mycotoxin.

The amount of adsorbed mycotoxin per unit weight biomass of adsorbents (Q_eq_) was first calculated according to the following equation [19,43,53].

Q_eq_ = [(C_0_−C_eq_)V]/m (2)

where Q_eq_ = quantity of mycotoxins absorbed by adsorbent (mg/g); C_0_ = concentration in the supernatant of adsorbent-free control groups (μg/mL); C_eq_ = residual mycotoxin concentration in the supernatant of adsorbent-treated groups at equilibrium (μg/mL); V = volume of solution (mL); and m = mass of adsorbent (g).

The calculated Q_eq_ values were then fitted into three adsorption isotherm models that have been frequently reported in the literature, namely, Hill, Langmuir, and Freundlich, [18,19,53,57] using OriginPro, 2022 (OriginLab Corporation, Northampton, MA, USA) (Table 4).

The goodness of fit was applied and evaluated by the coefficient of determination (R^2^), residual sum of squares (SS_RES_) and reduced chi-squared (χ^2^) to access the best-fitting model for each adsorption process [18,46,53]; lower χ^2^ and SS_RES_ or higher R^2^ are indicative of a better fit of each respective model. The best-fitting models were then selected for each mycotoxin–adsorbent interaction and used to obtain the parameters to predict a potential efficacy of sequestration (Table 4). For the adsorption processes that were best described by the Hill’s model, the binding potential was then calculated from the obtained parameters according to the following equation [64,71]:

Binding Potential = V_max_/K_d_ (3)

### 5.5. Adsorption Efficiency

The adsorption efficiency was calculated as follows as another endpoint to assess the adsorbent efficacy [20,54]:

Adsorption efficiency (%) = (C_0−_C_eq_)/C_0_ × 100 (4)

where C_0_ = concentration in the supernatant of the adsorbent-free control groups (μg/mL) and C_eq_ = residual mycotoxin concentration in the supernatant of adsorbent-treated groups at equilibrium (μg/mL).

### 5.6. Bioassay

#### 5.6.1. Cell Culture

The bovine mammary epithelial cell line (MAC-T) [72] was maintained in culture medium containing Dulbecco’s Modified Eagle Medium (DMEM) supplemented with 4.0 mmol/L L-glutamine, 10% heat-inactivated fetal bovine serum, 2.5% HEPES buffer, 1% penicillin/streptomycin (100 units/mL of penicillin and 100 g/mL of streptomycin) and 1 mM sodium pyruvate (Invitrogen, Thermo Fischer Scientific, Waltham, MA, USA), and it was passaged at 80% confluency by trypsinization using TrypLE™ (Gibco # 12605036, Thermo Fischer Scientific, Waltham, MA, USA) for the experiments. The cell culture was maintained in a humidified incubator at 37 °C with 5% CO_2_ as previously described [41].

#### 5.6.2. Cytotoxicity Assay

MAC-T cells were seeded at a density of 2 × 10^4^ cells per well in 96-well microplates [41,55]. A volume of 1 mL supernatant subsamples of DON (2 and 4 μg/mL), BEA (20 and 40 μg/mL), OTA (20 and 30 μg/mL), and CIT (50 and 68 μg/mL) preserved from the GIT incubation procedure (Section 2.2) were lyophilized using a freeze dryer (Harvest Right, Canada). The concentrations that were selected had the highest two cytotoxic concentrations reported in our previously published studies [41,55].

The lyophilized samples that contained mycotoxin residues were re-dissolved in 1 mL of cell culture medium as previously described with slight modifications in an attempt to reach the designated mycotoxin concentrations in the present work [73], and then they were administered to the cells for a 48 h exposure. At the end of mycotoxin exposure, cells in each well were incubated with 2 μmol/L of cell-permeant fluorescent dye, Calcein AM (Invitrogen, Thermo Fischer Scientific, Waltham, MA, USA), at room temperature for 45 min. The fluorescence intensity (FI) for each well was measured using a microplate reader at excitation 498 and emission 528 nm. The percentage of viable cells was calculated using the following formula according to the reference [41]:

Cell viability (%) = Mean (FI498 Treated cells−FI Blank)/Mean (FI498 Untreated cells−FIBlank) × 100(5)

where FI_498_ Treated cells was the FI obtained from mycotoxin-treated groups, FI_498_ Untreated cells was the FI obtained from groups without any mycotoxin treatment, FI Blank was the background signal resulting from Calcein AM-treated wells without cells, and the Mean was the average FI of three replicates. The cell viability of groups containing both mycotoxin and adsorbent was compared against that of adsorbent-free control groups.

### 5.7. Statistical Analysis

The goodness-of-fit statistic was obtained through model fitting outputs from OrginPro, 2022 (OriginLab Corporation, Northampton, MA, USA). A two-way ANOVA was performed to analyze adsorption percentage and cell viability data, and it was followed by Tukey and Dunnett post hoc analyses, respectively. The data are presented as mean ± SEM of three independent experiments conducted in triplicate, and *p* < 0.05 was considered statistically significant.

## Figures and Tables

**Table 1:** Evaluation of the goodness of fit of selected adsorption isotherm models toward the evaluation of the adsorption properties of three yeast cell wall-based adsorbents, namely yeast cell walls (YCW), yeast cell wall extract (YCWE), and postbiotic yeast cell wall-based blend (PYCW) toward DON, BEA, CIT, and OTA mycotoxins.

Mycotoxin	Adsorbent	Hill			Langmuir			Freundlich		
		R^2^	SS_RES_	Reduced χ^2^	R^2^	SS_RES_	Reduced χ^2^	R^2^	SS_RES_	Reduced χ^2^
**DON**	YCW	0.9154	0.446	0.030	nc			0.9154	0.446	0.028
	YCWE	0.8429	1.196	0.080	nc			0.7792	1.680	0.105
	PYCW	nc			0.3121	1.287	0.080	0.1685	1.556	0.097
										
BEA	YCW	0.9634	3.640	0.243	0.9577	4.211	0.263	0.8619	13.751	0.859
	YCWE	0.8150	33.208	2.214		nc		0.7766	40.082	2.505
	PYCW	0.8881	33.102	2.207	0.7142	84.584	5.286	0.5958	119.612	7.476
										
CIT	YCW	0.7649	4.878	0.325	0.7404	5.387	0.337	0.7090	6.039	0.377
	YCWE	0.9366	1.800	0.120	0.9307	1.967	0.123	0.9135	2.455	0.153
	PYCW	nc				nc		0.6361	15.133	0.946
								
OTA	YCW	nc			0.7388	0.446	0.028	0.7835	0.370	0.023
	YCWE	0.6874	0.973	0.065	nc			0.6874	0.973	0.061
	PYCW	nc			nc			0.7953	1.742	0.109

DON = deoxynivalenol; BEA = beauvericin; CIT = citrinin; OTA = ochratoxin A; R^2^ = coefficient of determination; SS_RES_ = residual sum of squares; Reduced χ^2^ = reduced chi-squared; nc = did not converge.

**Table 2:** Evaluation of best-fitting models and model parameter calculations for each of three yeast cell wall-based adsorbents, namely yeast cell walls (YCW), yeast cell wall extract (YCWE), and postbiotic yeast cell wall-based blend (PYCW) toward DON, BEA, CIT, and OTA mycotoxins.

Mycotoxin	Adsorbent	Best-Fitting Model	Parameters		Binding Potential
**DON**	YCW	Hill	Vmax (mg/g) ± SE	45.67 ± 11.174	0.258
			K_d_ (μg/mL) ± SE	176.80 ± 33.58	
			n ± SE	1.48 ± 0.749	
	YCWE	Hill	Vmax (mg/g) ± SE	1.75 ±0.170	0.188
			K_d_ (μg/mL) ± SE	9.31 ± 0.632	
			n ± SE	12.46 ± 1.258	
	PYCW	na	na	na	na
					
BEA	YCW	Hill	Vmax (mg/g) ± SE	5.91 ± 0.357	2.300
			K_d_ (μg/mL) ± SE	2.57 ± 0.520	
			n ± SE	1.30 ± 0.242	
	YCWE	Hill	Vmax (mg/g) ± SE	7.69 ± 1.913	1.376
			K_d_ (μg/mL) ± SE	5.59 ± 2.615	
			n ± SE	1.85 ± 0.979	
	PYCW	Hill	Vmax (mg/g) ± SE	8.80 ± 0.775	2.465
			K_d_ (μg/mL) ± SE	3.57 ± 0.186	
			n ± SE	9.33 ± 6.141	
					
CIT	YCW	Hill	Vmax (mg/g) ± SE	2.10 ± 0.309	0.230
			K_d_ (μg/mL) ± SE	9.13 ± 4.982	
			n ± SE	2.74 ± 2.387	
	YCWE	Hill	Vmax (mg/g) ± SE	3.86 ± 0.897	0.175
			K_d_ (μg/mL) ± SE	22.11 ± 9.316	
			n ± SE	1.41 ± 0.394	
	PYCW	Freundlich	K_f_ (μg/mL) ± SE	1.61E-05	na
			1/n ± SE	3.21 ± 1.6	
					
OTA	YCW	Freundlich	K_f_ (μg/mL) ± SE	0.18 ± 0.048	na
			1/n ± SE	0.48 ± 0.096	
	YCWE	Freundlich	K_f_ (μg/mL) ± SE	0.01 ± 0.018	na
			1/n ± SE	1.13 ± 0.403	
	PYCW	Freundlich	K_f_ (μg/mL) ± SE	0.06 ± 0.046	na
			1/n ± SE	1.11 ± 0.275	

DON = deoxynivalenol; BEA = beauvericin; CIT = citrinin; OTA = ochratoxin A; YCW = yeast cell wall; YCWE = yeast cell wall extract; PYCW = postbiotic yeast cell-wall-based blend; na = not applicable.

**Table 3:** Overall mean adsorption efficiency for three yeast cell-wall-based adsorbents (yeast cell walls (YCW), yeast cell wall extract (YCWE), and postbiotic yeast cell-wall-based blend (PYCW)) toward DON, BEA, CIT, and OTA mycotoxins.

Mycotoxin	Overall Mean Adsorption Efficiency (%) (Mean ± SEM)^1^		
	YCW	YCWE	PYCW
DON	18.45 ± 3.204 ^a^	21.72 ± 4.300 ^a^	33.46 ± 7.993 ^b^
BEA	73.37 ± 6.794 ^a^	61.00 ± 4.771 ^b^	70.15 ± 7.912 ^ab^
CIT	22.90 ± 1.151 ^a^	26.10 ± 1.661 ^ab^	35.04 ± 6.783 ^b^
OTA	32.28 ± 7.111 ^a^	23.55 ± 5.785 ^b^	36.86 ± 5.150 ^a^

DON = deoxynivalenol; BEA = beauvericin; CIT = citrinin; OTA = ochratoxin A. ^1^ Values are the mean ± SEM of three independent experiments. ^a–b^ Values labeled with different superscript letters in a row indicate significant differences between adsorbent treatments (*p* < 0.05).

**Table 4:** Equations and parameters used as adsorption isotherm models.

Adsorption Isotherm Model	Equation	Parameters	Reference
Hill’s	Q_eq_ = V_max_C_eq_^n^/(K_d_^n^ + C_eq_^n^)	V_max_ = maximum mycotoxin uptake K_d_ = dissociation constant per site (ug/mL) related to adsorption affinity n = Hill cooperativity coefficient of the binding interaction; minimum number of binding sties	[47,53,57]
Langmuir	Q_eq_ = V_max_K_L_C_eq_/(1 + K_L_C_eq_)	V_max_ = maximum mycotoxin uptake K_L_ = constant related to affinity of adsorption	
Freundlich	Q_eq_ = K_f_C_eq_^1/n^	K_f_ = constant indicating capacity of the adsorbent for the mycotoxin n = adsorption intensity	

## Data Availability

The datasets used and analyzed during the current study available from the corresponding author upon request.
